# Regional heterogeneity in violence and individual characteristics associated with recent transactional sex among Ugandan girls and young women: A national and regional analysis of data from the Violence Against Children and Youth Survey

**DOI:** 10.1371/journal.pone.0257030

**Published:** 2021-09-02

**Authors:** Caroline Stamatakis, Ashleigh Howard, Laura Chiang, Greta M. Massetti, Rose Apondi, Kirsten Stoebenau, Jennifer Hegle, Lydia Wasula, Pragna Patel

**Affiliations:** 1 National Center for Injury Prevention and Control, Centers for Disease Control and Prevention, Atlanta, Georgia, United States of America; 2 Center for Global Health, Centers for Disease Control and Prevention, Atlanta, Georgia, United States of America; 3 School of Public Health, University of Maryland, College Park, Maryland, United States of America; 4 Uganda Ministry of Gender, Labour and Social Development, Kampala, Uganda; Population Council, INDIA

## Abstract

**Objectives:**

This study assessed associations between recent transactional sex (TS) and potential determinants and variations in patterns across two geographic regions with high HIV burden compared to the rest of Uganda, among adolescent girls and young women (AGYW).

**Methods:**

In 2015, a nationally representative cross-sectional household survey was conducted in Uganda. A stratified multi-stage cluster sample design produced nationally representative estimates and sub-national estimates for AGYW in two high HIV burden regions, DREAMS Central 1 (Bukomansimbi, Ssembabule, and Rakai districts) and DREAMS Central 2 (Mubende, Mityana, Gomba, and Mukono districts), and the rest of Uganda. To identify associations between recent TS (defined as sex in the past 12 months in exchange for material support or help) and risk factors, multivariable logistic regressions were conducted. Interaction terms assessed the associations between violence and recent TS across geographic regions.

**Results:**

Nationally, 14.2% of sexually active AGYW engaged in recent TS. Region-specific significant associations emerged between recent TS and marriage, family wealth, friendship, orphanhood, and sexual debut. In DREAMS Central 1 and 2, AGYW who witnessed violence in the home or community, or experienced sexual, physical, or emotional violence had higher odds of recent TS than AGYW who did not experience that form of violence (adjusted odds ratio ranged between 2.10 (95% CI, 1.07, 4.13) and 8.25 (95% CI, 3.40, 20.06)). The magnitude of association between recent TS and types of violence varied by region.

**Conclusions:**

Violence is strongly and consistently associated with recent TS, and patterns in prevalence and risk factors vary across regions in Uganda. Given the high risk of HIV association with recent TS, HIV epidemic control efforts may benefit from focus on comprehensive violence prevention and target persons who engage in TS. Comprehensive HIV prevention programming aimed at keeping AGYW HIV-negative should incorporate prevention of violence and TS as key components to facilitate HIV epidemic control in this vulnerable population.

## Introduction

Uganda has a generalized HIV epidemic with an estimated prevalence of 6.2% among 15–64 year olds and an annual incidence of 0.4% among adults, resulting in approximately 73,000 new cases each year [1]. As in many sub-Saharan African countries, adolescent girls and young women (AGYW) in Uganda are disproportionately impacted, with an HIV prevalence four times higher among older adolescent females than their same-age male peers. Identifying and responding to the underlying factors that put AGYW at greater risk of HIV is critically important to epidemic control and much research, programming, and policy has begun to unpack and address the marked vulnerability of AGYW. Factors ranging from the structural to the individual levels contribute to AGYW’s heightened risk of HIV including poverty, gender inequality, education, early sexual debut and early pregnancy, and sexual risk behaviors such as transactional sex (TS) [2–4]. Both a systematic review and meta-analysis have demonstrated an association between TS and HIV, and longitudinal studies have indicated a causal link between TS and incident HIV among AGYW [5–8]. Given this evidence, it is important to understand the factors associated with TS and the practice of TS, as this has demonstrated implications for HIV prevention programming, including improved intervention targeting. This is particularly relevant for Uganda given the high prevalence of both TS and HIV among AGYW [1,9].

For this research, we consider TS as a noncommercial sexual exchange practice structured by social and economic gender inequality. Transactional sex occurs within the context of a sexual relationship or experience and is based on an implicit assumption that sex will be exchanged for material support or some other benefit [10]. Girls and women engage in TS for a variety of reasons that can range from securing basic needs (survival sex) to improving their social status or peer approval, often in part through acquiring luxury items [11,12]. Emerging evidence has begun to identify important factors at the interpersonal and individual levels that help to explain AGYW’s heightened risk to HIV through engagement in TS. First, there are important linkages between violence, TS, and HIV among AGYW [13,14]. TS is associated with previous experience of abuse or violence, including sexual coercion [15]; emotional, physical or sexual abuse [15]; and intimate partner violence, including physical or sexual violence [16,17]. The link between intimate partner violence and TS has been explained in the South African context through roles and expectations attached to ideals about “dominant” masculinity; these ideals include the expectations that men will both “provide for” and “control” their female partners [18,19]. Relatedly, TS is associated with low sexual relationship power for women [20], and there is a social expectation that men should hold sexual decision-making power in relationships where they provide financial support [21]. In addition, TS can put AGYW at increased risk for HIV due to having more and older sexual partners as well as partner concurrency [18,22,23], age-disparate TS relationships [24,25], lower condom use [26], and increased alcohol misuse [27].

While there is considerable research on determinants of (or motivations for AGYW to engage in) TS, this paper addresses the geographic heterogeneity of this sexual practice within Uganda and seeks to understand how risk for TS may vary regionally. Understanding these regional differences is essential in understanding risk for HIV and targeting epidemic control efforts. This study seeks to assess variations in patterns of potential determinants for recent TS across two geographic regions with high HIV burden compared to the remaining geographic regions of Uganda among AGYW (13–24 years old). The sub-national geographic (‘regional’) data presented here were collected in two priority areas, ‘Central 1’ and ‘Central 2’, that were designated as locations for the HIV prevention program known as DREAMS (Determined, Resilient, Empowered, AIDS-free, Mentored, and Safe). DREAMS is a public-private partnership funded by the U.S President’s Emergency Plan for AIDS Relief (PEPFAR) that aims to reduce new HIV infections in AGYW through evidence-based interventions that address structural drivers related to the HIV risks specific to AGYW. The DREAMS partnership’s layered intervention approach to HIV prevention aims to develop resiliency and promote healthy choices among AGYW. DREAMS programming targets AGYW, their families and their communities through parenting and community violence prevention programs and also DREAMS programming focuses on preventing HIV and violence among AGYW through social asset building, which includes building peer networks, keeping girls in school, sexual risk reduction, and providing financial independence [2]. Beyond the issue of high HIV prevalence, the districts in DREAMS Central 1 were prioritized for HIV prevention among AGYW due to risks associated with its border location, fishing communities, and truck driver population. A higher HIV prevalence and increased rate of HIV risk behaviors, including TS [28], are particularly well documented in fishing communities, such as those in Central 1 and are attributed to the transient population, gender inequality, and HIV fatalism [29]. The districts in DREAMS Central 2 were prioritized based on HIV risk factors associated with trading hubs and major transportation routes, also well-documented in the literature as drivers of HIV [30,31].

As identified in prior studies, we first hypothesized that characteristics often associated with vulnerability, including poverty, low educational attainment, orphan status, and low social support would be associated with TS, along with adverse experiences, such as witnessing violence in the home or community in childhood and lifetime sexual, physical, or emotional violence victimization. We further expected that the specific characteristics, such as those associated with greater risk of HIV infection, to be more strongly associated with TS in the DREAMS regions than the rest of Uganda given complex contextual differences in the HIV epidemic in Uganda’s Central 1 and Central 2 regions.

## Materials and methods

### Background of Uganda VACS and objectives

In 2015, the Uganda Ministry of Gender, Labour, and Social Development, the Uganda Bureau of Statistics, the U.S. Centers for Disease Control and Prevention (CDC), U.S. Agency for International Development (USAID), UNICEF Uganda, Makerere University School of Public Health, and The ChildFund International Consortium, including The AfriChild Centre for Excellence, worked in partnership to implement the Uganda Violence Against Children and Youth Survey (VACS). The purpose of the Uganda VACS was to produce nationally representative and regional estimates on the burden and consequences of sexual, physical, and emotional violence. VACS are national, cross-sectional surveys of children and youth age 13–24 years [32].

### Sampling and DREAMS settings

**Fig 1:**
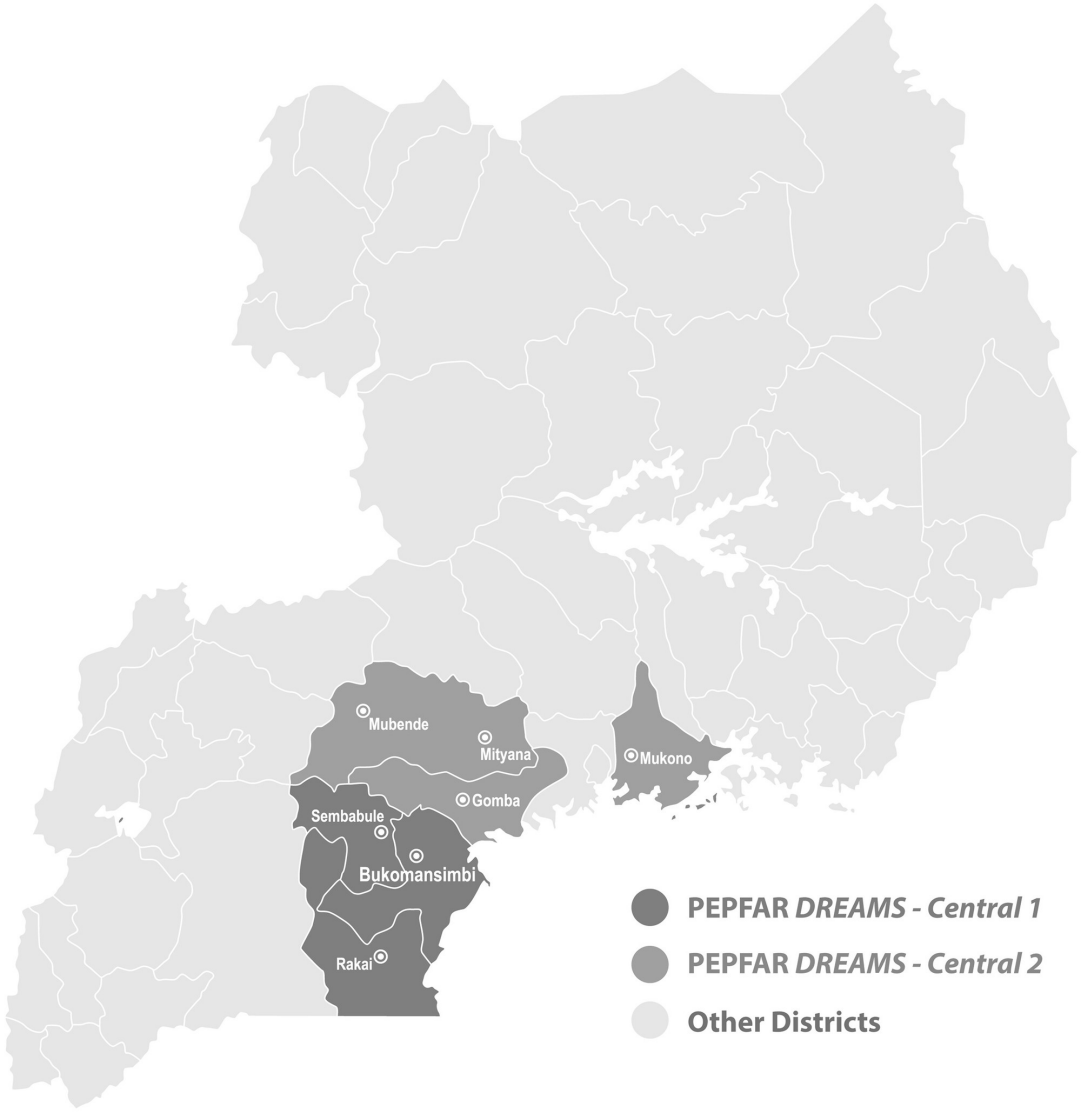
Uganda PEPFAR DREAMS clusters.

A geographically clustered, multi-stage sample design was used to produce nationally representative estimates of violence. A split sample approach selected separate enumeration areas for males and females in order to protect privacy, confidentiality, and safety of participants; however, only females were included in this analysis. During the first sampling stage, 368 female enumeration areas were selected using probability proportional to size. During the second stage, 25 households were systematically sampled in each enumeration area, and in the third stage, one eligible female was randomly selected per household and included in the sample, yielding a sample size of 3,159 completed female interviews. A description of the sample size and oversampling in each of the regions can be found in the Uganda VACS Report [9]. The overall response rate for females was 76.6%, with 3,970 females selected, 56 refusals, 620 not available, 7 incomplete interviews excluded from analysis, and 128 who were incapacitated/had language issues. Oversampling for AGYW in two regions, DREAMS Central 1 and DREAMS Central 2, yielded sub-national estimates for these regions. DREAMS Central 1 included Bukomansimbi, Ssembabule, and Rakai districts and DREAMS Central 2 included Mubende, Mityana, Gomba, and Mukono districts (Fig 1). A third DREAMS region was oversampled but is not presented separately due to a small number of females reporting TS in this area. All remaining enumeration areas (including the third DREAMS region and those regions not included in the DREAMS 1 and DREAMS 2 regions) were combined into Other Districts for the sub-national analyses.

### Eligibility criteria

Eligibility criteria included 13–24 years of age at the time of the survey and speaking one of the survey languages: English, Ateso-Karamajong, Luganda, Lugbara, Luo, Swahili, Runyankole-Rukiga, or Runyoro-Rutoro. AGYW who lived in institutions rather than households or who had a physical or cognitive disability that would interfere with participating in the survey were ineligible and not included in the study.

### Response plan and IRB review/ethics

The study protocol was independently reviewed and approved by the Makerere University College of Health Sciences ethics review committee, the Uganda National Council for Science and Technology (UNCST) review board, and the CDC Institutional Review Board. Prior to participation in the study, informed consent was obtained for 18-24-year-old participants. Informed consent by a parent or guardian and participant informed assent were obtained for the 13-17-year-old participants. Informed consent and assent were obtained verbally and documented through electronic signature in the netbooks. The oral consent/assent process was approved by each ethics institution during the independent review process. Ugandan survey teams trained by CDC and Uganda partners administered household face-to-face interviews and recorded participant responses on netbook computers [33]. Counseling and referral services were provided to participants who met specific criteria or requested help and participants who self-reported a positive HIV status were referred to the nearest government clinic for services. The study followed World Health Organization recommendations on ethics and safety in studies of violence against women [34].

### Weighted data for representative results

Survey weights were applied to the Uganda VACS data to yield nationally representative estimates and sub-national estimates for DREAMS 1, DREAMS 2, and Other Districts.

### Questionnaire

The VACS questionnaire was developed by a group of violence subject matter experts by drawing from the literature and including validated measures when available. The questionnaire was then adapted to the Ugandan context by Ugandan stakeholders. Prior to the survey in Uganda, the VACS questionnaire was cognitively tested to ensure that the VACS questions capture the intended content. Prior to Uganda VACS data collection, a pilot test of all data collection instruments and protocols was conducted. The Uganda VACS questionnaire can be accessed at the Together for Girls website (togetherforgirls.org).

### Variables

The outcome variable, recent TS, was asked among participants who answered yes to “*Have you had sex in the past 12 months*?” (n = 1,593) and assessed through the question, “*In the last 12 months*, *have you had sex with this person because they provided you with material support or help in any other way*? *Material support means helping you to pay for things*, *or giving you gifts or things such as food*, *school fees or money*.” This question was asked for the three most recent partners in the past 12 months. Sex was defined as vaginal, oral, or anal sex. Because the field and this study defines TS as being between non-married partners [10], where partner data were not missing, females who only engaged in TS with spouses were removed from analysis (n = 21 removed). Females who had a TS partner other than a spouse and those who had multiple TS partners where one or more partners was a non-spouse, were included in the analysis (n = 203). Females who engaged in TS in the past 12 months were also asked their age at first experience of TS, their relationship to their three most recent TS partners within the past 12 months (current or ex-romantic partner, current or ex-spouse, friend, and other), age of most recent TS partner (less than five years older and five or more years older), material goods provided by the most recent TS partner (money, food, school fees or supplies, favors or gifts, shelter or protection, and other), and HIV testing history and result.

Sociodemographic factors in these analyses include age, ever married or lived with someone as if married, child marriage, mean age at first marriage or cohabitation, family wealth, ever pregnant, highest level of school completion, orphanhood, food insecurity in the household, having a close or very close relationship with mother, having talked to friends about important things, early sexual debut, and ever tested for HIV. Violence and violence-related experiences included childhood witnessing of violence in the home or community and lifetime experiences of sexual, physical, and emotional violence. Sociodemographic factors, violence, and violence-related experiences are defined in Table 1.

**Table 1:** Definitions of sociodemographic factors and violence and violence-related variables.

Variable	Definition
Age	Age at the time of the survey Categorized as 13–17 or 18–24
Ever married	Ever married or lived with someone as if married
Child marriage	Ever married or lived with someone as if married before age 18
Mean age at first marriage	Mean age at first marriage or cohabitation (in years)
Ever pregnant	Ever been pregnant
Family wealth	Calculated using the Uganda Simple Poverty Scorecard [35] Categorized as: low, middle, and highest tertiles
Highest level of school completion	Completed primary school or less or attended or completed secondary school or higher
Orphanhood	Single or double orphan prior to age 18
Food insecurity	Food insecurity in the household was defined as answering yes to either of the following questions: Did you ever cut the size of the meals of child(ren) living in your household because there was not enough food or money? Did the child(ren) living in your household ever skip meals because there was not enough food or money?
Close or very close with mother	Close or very close with mother was defined as answering very close or close to the following question: How close do you/did you feel to your biological mother? Would you say very close, close, not close, or never had a relationship with her?
Talked with friends about important things	Talked with friends about important things was defined as answering a lot or some to the following question: How much do you talk to friends about important things: a lot, some, not too much, not at all?
Early sexual debut	First sex was before age 16
Ever tested for HIV	Ever tested for HIV was defined as answering yes to the following question: Have you ever been tested for HIV?
Witnessing violence in the home in childhood	Witnessing violence in the home in childhood was defined as answering "once", "few", or "many" to either of the following questions: *For respondents 13–17*: *At any time in your life*: *For respondents 18–24*: *Before the age of 18*: 1) How many times did you see or hear your parent punched, kicked or beaten up by your other parent, or their boyfriend or girlfriend? Never, once, a few times or many times? 2) How many times did you see or hear a parent punch, kick, or beat your brothers or sisters? Never, once, a few times, many times, or I have no brothers or sisters?
Witnessing violence in the community in childhood	Witnessing violence in the community in childhood was defined as answering "once", "few", or "many" to the following question: *For respondents 13–17*: *At any time in your life*: *For respondents 18–24*: *Before the age of 18*: 1) Outside of your home and family environment, how many times did you see anyone get attacked? Never, once, a few times, or many times?
Lifetime sexual violence	Lifetime sexual violence was defined as answering "yes" to any of the following questions: 1) Has anyone ever touched you in a sexual way without you wanting to, but did not try and force you to have sex? Touching in a sexual way without permission includes fondling, pinching, grabbing, or touching you on or around your sexual body parts. 2) Has anyone ever tried to make you have sex against your will but did not succeed? They might have tried to physically force you to have sex or they might have tried to pressure you have sex through harassment, threats and tricks. 3) Has anyone ever physically forced you to have sex and did succeed? 4) Has anyone ever pressured you to have sex, through harassment, threats or tricks and did succeed?
Lifetime physical violence	Lifetime physical violence was defined as answering "yes" to A, B, or C for any of the following questions: 1) Has a current or previous romantic partner, boyfriend or husband ever: 2) Has a person your own age ever: 3) Has a parent, adult caregiver, or other adult relative ever: 4) Has an adult in your community, such as teachers, police, employers, religious or community leaders, neighbors, or other adults you don’t know ever: A. punched, kicked, whipped, or beat you with an object? B. strangled, suffocated, tried to drown you, or burned you intentionally? C. used or threatened you with a knife, gun or other weapon?
Lifetime emotional violence	Lifetime emotional violence was defined as answering "yes" to A, B, or C: 1) Has a parent, adult caregiver or other adult relative ever: A. told you that you were not loved, or did not deserve to be loved? B. said they wished you had never been born or were dead? C. ever ridiculed you or put you down, for example said that you were stupid or useless?

### Statistical analysis

Descriptive analyses among sexually active AGYW ages 13–24 years assessed prevalence of sociodemographic factors, recent TS, and individual characteristics of AGYW who engaged in recent TS. To identify associations between recent TS and risk factors, univariate and multivariable logistic regressions were conducted. Variables that were significant at the p-value < 0.2 level in the univariate models were entered into multivariable logistic regression models. Control variables: sexual debut, ever married, family wealth, education, and ever pregnant, were chosen to be included in the multivariable models with a combination of identification as predictors of TS in prior studies [13,15] and had a p-value of less than.05 in one or more regions. Covariables in each model were limited to six variables. The cut-off was determined as a function of sample size using the rule of ten events, as not to over-fit the models. Interaction terms between region and types of violence assessed the associations between violence and recent TS within geographic regions (DREAMS Central 1, DREAMS Central 2, and Other Districts) and were included in separate unadjusted and adjusted models. Unweighted n’s, weighted percentages, weighted means, 95% confidence intervals (CIs), unadjusted odds ratios (ORs), adjusted ORs, and p-values were calculated using SAS 9.4 (SAS Institute Inc., Cary, North Carolina, USA). All calculations accounted for the complex survey design using the SURVEYFREQ procedure which includes STRATA, CLUSTER, and WEIGHT statements to account for design strata, clusters, and sampling weights.

## Results

Among Ugandan AGYW who had sex in the past 12 months (50.8% of all Uganda AGYW), the majority had been married or lived with someone as if married (82.2%) with more than a third (36.0%) being married before the age of 18, indicated in Table 2. Table 2 also shows several other demographic information that are briefly mentioned. The mean age at first marriage was 18.0 years. Most AGYW had ever been pregnant: 73.8% at the national level, 84.8% in DREAMS Central 1, 82.2% in DREAMS Central 2, and 73.1% in Other Districts. Nationally, approximately a third of AGYW were currently attending or completed secondary school or higher (35.9%), and 45.2% were in the highest tertile for family wealth. Almost two thirds (66.1%) of AGYW nationally had witnessed violence at home, 51.3% witnessed violence in the community, 47.7% experienced sexual violence, 72.4% experienced physical violence, and 44.4% experienced emotional violence. At the national level, 14.2% of sexually active AGYW engaged in recent TS (TS in the past 12 months). The prevalence of recent TS was significantly higher in DREAMS Central 1 (20.5%) compared to Other Districts (13.8%; *p* = 0.047). Recent TS prevalence was 19.2% in DREAMS Central 2.

**Table 2:** Individual characteristics and prevalence of recent transactional sex among sexually active^a^ Ugandan adolescent girls and young women ages 13–24 years old.

Individual Characteristics	National	DREAMS Central 1	DREAMS Central 2	Other Districts
	N = 1593% (95% CI)	N = 290% (95% CI)	N = 309% (95% CI)	N = 994% (95% CI)
**Age (years)**				
13–17	11.8 (9.0–14.5)	9.1 (5.7–12.6)	12.2 (8.7–15.7)	11.8 (8.9–14.8)
18–24	88.2 (85.5–91.0)	90.9 (87.4–94.3)	87.8 (84.3–91.3)	88.2 (85.2–91.1)
**Marriage**				
Ever married or lived with someone as if married	82.2 (78.6–85.8)	88.9 (85.1–92.7)	85.2 (80.9–89.4)	81.9 (78.0–85.7)
Child marriage (married before age 18)	36.0 (32.0–39.9)	37.3 (32.4–42.3)	31.9 (26.9–36.9)	36.1 (31.9–40.3)
Mean age at first marriage	18.0 years (17.5–18.4)	17.9 years (17.6–18.1)	18.1 years (17.9–18.4)	17.9 years (17.4–18.4)
**Pregnancy**				
Ever pregnant	73.8 (69.3–78.3)	84.8 (80.0–89.6)	82.2 (78.3–86.1)	73.1 (68.3–77.9)
**Highest level of school**				
Completed primary school or less	64.1 (58.2–70.1)	67.3 (59.0–75.7)	60.1 (54.1–66.1)	64.3 (57.9–70.7)
Attending/completed secondary school or higher	35.9 (29.9–41.8)	32.7 (24.3–41.0)	39.9 (33.9–45.9)	35.7 (29.3–42.1)
**Family Wealth**				
Low	27.7 (21.8–33.6)	13.8 (8.6–19.0)	12.8 (8.9–16.7)	28.8 (22.4–35.1)
Middle	27.0 (22.1–32.0)	32.7 (25.9–39.6)	30.0 (24.0–36.0)	26.8 (21.5–32.1)
Highest	45.2 (38.1–52.3)	53.4 (44.5–62.4)	57.2 (49.7–64.7)	44.5 (36.8–52.1)
**Violence**				
Witnessed violence in the home prevalence	66.1 (61.3–70.8)	69.6 (62.4–76.8)	66.3 (60.9–71.6)	66.0 (60.9–71.0)
Witnessed violence in the community prevalence	51.3 (45.9–56.7)	51.3 (45.1–57.5)	46.2 (38.2–54.1)	51.5 (45.7–57.3)
Sexual violence prevalence	47.7 (43.1–52.2)	58.9 (51.7–66.2)	62.0 (56.6–67.4)	46.7 (41.9–51.6)
Physical violence prevalence	72.4 (68.0–76.8)	76.8 (68.8–84.9)	77.5 (72.6–82.3)	72.1 (67.4–76.8)
Emotional violence prevalence	44.4 (39.4–49.4)	43.0 (35.5–50.5)	35.4 (30.4–40.5)	44.8 (39.5–50.2)
**Transactional Sex**				
Prevalence of recent TS	14.2 (10.7–17.6)	20.5 (14.7–26.2)	19.2 (14.2–24.1)	13.8 (10.1–17.5)

Note: CI = Confidence interval; Recent TS = transactional sex in the past 12 months.

Respondents with missing data or who answered “don’t know” or refused are excluded from the denominators.

^a^Sexually active is defined as having had vaginal, oral, or anal sex in the past 12 months.

Table 3 depicts several characteristics of TS in this population. Among AGYW who had recent TS, the mean age at first experience of TS nationally was 17.9 years old. The majority of the recent TS occurred with current or ex-romantic partners (79.2%) or current or ex-spouses (24.6%), though those who reported transactional sex with a spouse also reported transactional sex with someone else. Those who only had transactional sex with a spouse in the last 12 months were removed from the analysis. TS with friends (6.5%) and others (2.0%) was less common. Similarly, in DREAMS Central 1, DREAMS Central 2, and Other Districts the majority of recent TS occurred with current or ex-romantic partners (60.0%, 72.9%, 80.2%, respectively) or current or ex-spouses (34.5%, 32.2%, 23.8%, respectively). Nationally, fewer than half (43.8%) of the most recent TS partners were five or more years older. Material goods provided by the most recent TS partner included money (77.7%), favors or gifts (52.9%), food (40.0%), school fees or supplies (16.6%), shelter or protection (6.0%) and other (2.3%). Likewise, in DREAMS Central 1, DREAMS Central 2, and Other Districts the majority of material good provided by the most recent TS partner was money (87.1%, 95.0%, 76.2%, respectively). Among AGYW who had recent TS, nationally 1.3% self-reported that they were living with HIV, 4.3% in DREAMS Central 1, 5.6% in DREAMS Central 2, and 0.9% in Other Districts.

**Table 3:** Characterization of recent transactional sex, among Ugandan adolescent girls and young women who engaged in recent transactional sex, ages 13–24 years old.

Characteristics of recent TS^a^	National	DREAMS Central 1	DREAMS Central 2	Other Districts
	N = 203% (95%CI)	N = 60% (95%CI)	N = 60% (95%CI)	N = 83% (95%CI)
Mean age at first experience of TS	17.9 years (17.4–18.4)	17.6 years (16.9–18.2)	17.4 years (16.8–18.1)	17.9 years(17.4–18.5)
Relationship to recent TS partner**				
Current or ex-romantic partner	79.2 (69.8–88.5)	60.0 (46.1–73.9)	72.9 (60.1–85.7)	80.2 (70.1–90.4)
Current or ex-spouse	24.6 (13.0–36.3)	34.5 (22.2–46.9)	32.2 (19.3–45.1)	23.8 (11.1–36.5)
Friend	6.5 (1.2–11.8)*	13.8 (4.1–23.4)*	4.9 (0.0–10.2)*	6.3 (0.5–12.2)*
Other^b^	2.0 (0.0–5.1)*	7.7 (0.0–15.8)*	3.3 (0.0–7.9)*	1.8 (0.0–5.1)*
Age of most recent TS partner				
Less than 5 years older	56.2 (42.6–69.8)	55.4 (43.5–67.3)	49.0 (35.4–62.5)	56.7 (41.8–71.7)
5 or more years older	43.8 (30.2–57.4)	44.6 (32.7–56.5)	51.0 (37.5–64.6)	43.3 (28.3–58.2)
Material goods provided by most recent TS partner**				
Money	77.7 (66.5–88.9)	87.1 (77.5–96.6)	95.0 (89.8–100.0)	76.2 (63.9–88.6)
Favors or gifts	52.9 (39.5–66.3)	54.2 (41.5–67.0)	58.2 (44.4–72.1)	52.5 (37.8–67.2)
Food	40.0 (26.6–53.4)	46.4 (31.7–61.0)	32.7 (16.7–48.7)	40.3 (25.6–55.0)
School fees or supplies	16.6 (6.5–26.8)*	1.6 (0.0–4.9)*	5.0 (0.0–12.5)*	17.9 (6.7–29.0)*
Shelter or protection	6.0 (1.5–10.6)*	21.0 (9.1–32.9)	12.4 (4.4–20.4)*	5.1 (0.2–10.0)*
Other^c^	2.3 (0.0–5.3)*	0.0	3.7 (0.0–10.7)*	2.2 (0.0–5.5)*
Self-reported HIV positive status	1.3 (0.0–3.1)*	4.3 (0.0–10.6)*	5.6 (0.0–12.9)*	0.9 (0.0–2.8)*

Note: TS = transactional sex; CI = Confidence interval.

Respondents with missing data or who answered “don’t know” or refused are excluded from the denominators.

^a^Recent TS is defined as transactional sex in the past 12 months.

^b^Other relationship to partner includes: Teacher, neighbor, stranger, and other.

^c^Other material goods include: Employment and transportation.

*Unreliable estimate (RSE is > 30%), result should be interpreted with caution.

**Sums to >100% because categories are not mutually exclusive.

Table 4 includes results of logistic regressions assessing relationships between risk factors and recent TS by geographic region. In the unadjusted models, being married or living with someone as if married (cohabiting) and talking to friends about important things were associated with lower prevalence of TS in DREAMS Central 1. Early sexual debut, witnessing violence in the home and community in childhood, and sexual, physical, and emotional violence were all positively associated with recent TS (unadjusted ORs, CIs, and *p*-values for univariate associations are in Table 4). In DREAMS Central 2, TS was significantly positively associated with early sexual debut, negatively associated with ever tested for HIV, and positively associated with witnessing violence in the home and in the community, sexual, physical, and emotional violence. In Other Districts, TS was significantly negatively associated with being married or cohabiting, being in the low family wealth tertile, ever being pregnant, and positively associated with sexual and emotional violence.

**Table 4:** Multivariable comparisons between sociodemographic factors, violence, and violence related experiences and recent transactional sex^a^ among sexually active^b^ Ugandan adolescent girls and young women ages 13–24 years old.

	DREAMS Central 1 n = 276				DREAMS Central 2 n = 284				Other District n = 955			
	Unadjusted OR (95% CI)	P-value	Adjusted OR* (95% CI)	P-value	Unadjusted OR (95% CI)	P-value	Adjusted OR* (95% CI)	P-value	Unadjusted OR (95% CI)	P-value	Adjusted OR* (95% CI)	P-value
**Sociodemographic factors**												
**Marriage**												
Ever married or lived with someone as if married	**0.42 (0.20–0.86)** ^c^	**0.0188**	0.39 (0.12–1.29)	0.1221	0.61 (0.29–1.31)	0.2056			**0.24 (0.11–0.52)**	**0.0004**	**0.19 (0.07–0.51)**	**0.0011**
Child marriage (married before age 18)	0.85 (0.52–1.40)	0.5243			1.63 (0.87–3.07)	0.1267	1.41 (0.67–2.97)	0.36	0.56 (0.30–1.01)	0.0722	0.97 (0.50–1.90)	0.9263
**Family Wealth**												
Low	0.67 (0.27–1.71)	0.4016			0.97 (0.34–2.81)	0.957			**0.45 (0.21–0.95)**	**0.0363**	**0.38 (0.18–0.80)**	**0.0106**
Middle	0.66 (0.35–1.26)	0.2015			1.53 (0.81–2.89)	0.1859	1.58 (0.78–3.20)	0.1973	0.68 (0.31–1.49)	0.3347		
Highest	ref	-			ref	-			ref	-		
**Highest level of school**												
Completed primary school or less	0.73 (0.37–1.42)	0.3443			1.62 (0.85–3.09)	0.1432	1.57 (0.74–3.35)	0.2384	0.72 (0.36–1.42)	0.3435		
Attending/completed secondary school or higher	ref	-			ref	-			ref	-		
**Orphanhood**												
Single or double orphan prior to age 18	0.91 (0.44–1.92)	0.8099			0.70 (0.36–1.37)	0.2909			1.74 (0.97–3.12)	0.0638	**1.87 (1.04–3.37)**	**0.0373**
**Food Access**												
Food insecurity in household	1.64 (0.78–3.46)	0.1898	1.97 (0.86–4.50)	0.1061	1.40 (0.65–3.02)	0.3923			0.83 (0.45–1.53)	0.5528		
**Relationships**												
Close or very close with mother	1.09 (0.43–2.74)	0.8586			1.27 (0.48–3.39)	0.631			0.77 (0.33–1.80)	0.539		
Talked to friends about important things (a lot or a little)	**0.46 (0.25–0.86)**	**0.0149**	**0.41 (0.21–0.80)**	**0.0095**	0.74 (0.40–1.37)	0.3282			1.33 (0.70–2.52)	0.3799		
**Pregnancy**												
Ever pregnant	0.61 (0.30–1.25)	0.1719	0.84 (0.27–266)	0.7663	0.63 (0.28–1.41)	0.2606			**0.49 (0.25–0.96)**	**0.0384**	1.27 (0.56–2.91)	0.1567
**Sexual Debut**												
Early sexual debut (before age 16)	**2.42 (1.27–4.60)**	**0.0077**	**2.54 (1.33–4.83)**	**0.0052**	**2.58 (1.21–5.49)**	**0.0148**	**2.71 (1.24–5.96)**	**0.0135**	1.19 (0.58–2.41)	0.635		
**HIV Testing**												
Ever tested for HIV	0.65 (0.25–1.73)	0.3839			**0.26 (0.08–0.80)**	**0.0193**	0.32 (0.09–1.21)	0.0915	0.48 (0.16–1.45)	0.1903	0.72 (0.23–2.21)	0.5617
**Violence and violence-related experiences**												
Witnessed violence in the home	**2.32 (1.17–4.59)**	**0.0164**	**2.10 (1.07–4.13)**	**0.0313**	**2.44 (1.29–4.64)**	**0.007**	**2.52 (1.29–4.92)**	**0.0073**	1.23 (0.61–2.49)	0.5572		
Witnessed violence in the community	**3.61 (1.90–6.86)**	**0.0002**	**3.71 (1.90–7.23)**	**0.0002**	**3.52 (1.76–7.05)**	**0.0005**	**3.43 (1.62–7.26)**	**0.0016**	1.08 (0.59–1.97)	0.8039		
Sexual violence	**3.53 (1.85–6.76)**	**0.0002**	**2.94 (1.49–5.81)**	**0.0023**	**7.80 (3.35–18.15)**	**< .0001**	**8.25 (3.40–20.06)**	**< .0001**	**1.92 (1.03–3.58)**	**0.0399**	1.89 (1.00–3.56)	0.0487
Physical violence	**8.29 (2.37–28.98)**	**0.0012**	**6.57 (1.82–23.76)**	**0.0047**	**2.67 (1.20–5.94)**	**0.0167**	2.37 (0.99–5.70)	0.0534	1.30 (0.61–2.80)	0.4955		
Emotional violence	**3.57 (1.93–6.63)**	**0.0001**	**4.01 (2.03–7.90)**	**0.0001**	**3.21 (1.57–6.55)**	**0.0017**	**2.92 (1.37–6.24)**	**0.0062**	**2.23 (1.15–4.34)**	**0.0184**	**2.28 (1.13–4.58)**	**0.0211**

CI = confidence interval.

^a^Recent transactional sex is defined as transactional sex in the past 12 months.

^b^Sexually active is defined as having sex (vaginal, oral, or anal sex) in the past 12 months.

^c^Bolded results have odds ratio p-values less than 0.05.

*OR Adjusted for: Early sexual debut, ever married, family wealth, education, and ever pregnant.

Note: Only variables that were significant at the *p* < 0.2 level in the unadjusted logistic regression models were entered into adjusted models.

After adjusting for early sexual debut, marriage, family wealth, education, and pregnancy, significant associations between recent TS and risk factors varied across DREAMS regions and Other Districts. In DREAMS Central 1, AGYW who talked to friends about important things had decreased odds of recent TS (AOR = 0.41, 95% CI: 0.21, 0.80), and those who had early sexual debut had increased odds of recent TS (AOR = 2.54, 95% CI: 1.33, 4.83). In DREAMS Central 2, AGYW with early sexual debut had 2.71 greater odds of engaging in recent TS (95% CI: 1.24, 5.96) than AGYW whose first sex was at age 16 or later. In Other Districts, AGYW who had ever been married or lived with someone as if married (AOR = 0.19, 95% CI: 0.07, 0.51) and had low family wealth (AOR = 0.38, 95% CI: 0.18, 0.80) had decreased odds of recent TS, and those who had single or double orphanhood prior to age 18 (AOR = 1.87, 95% CI: 1.04, 3.37) had increased odds of recent TS.

There were consistent associations between violence and recent TS in the DREAMS regions. In both DREAMS Central 1 and 2, AGYW who experienced any form of violence (witnessing violence in the home or community in childhood, sexual violence, physical violence, and emotional violence) had higher odds of recent TS than AGYW who did not experience those forms of violence, with AORs ranging from 2.10 (95% CI, 1.07, 4.13) for witnessing violence in the home in DREAMS Central 1 to 8.25 (95% CI, 3.40, 20.06) for sexual violence in DREAMS Central 2. The only exception was physical violence in DREAMS Central 2, which had an AOR of 2.37 (95% CI, 0.99, 5.70) and a *p*-value of 0.0534. In Other Districts, AGYW who experienced emotional violence had higher odds of recent TS (AOR = 2.28, 95% CI: 1.13, 4.58) compared to those who did not experience emotional violence.

The magnitude of association between violence and recent TS varied by geographic area as shown in Table 5. In DREAMS Central 1 and DREAMS Central 2, those who witnessed violence in the home compared to those who did not, had higher odds of engaging in recent TS (DREAMS Central 1 AOR = 2.21, 95% CI: 1.12, 4.34; DREAMS Central 2 AOR = 2.72, 95% CI: 1.39, 5.30) and the odds were significantly higher than the odds in Other Districts (DREAMS Central 1 vs Other Districts *p* = 0.0297; DREAMS Central 2 vs Other Districts *p* = 0.0371). A similar pattern was observed for witnessing violence in the community (DREAMS Central 1 AOR = 3.54, 95% CI: 1.81, 6.96; DREAMS Central 2 AOR = 3.41, 95% CI: 1.62, 7.19), and there were significantly higher odds in DREAMS Central 1 and 2 than the odds in Other Districts (DREAMS Central 1 vs Other Districts *p* = 0.0087; DREAMS Central 2 vs Other Districts *p* = 0.0094). AGYW in all regions who experienced sexual violence compared to those who did not, had higher odds of engaging in recent TS (DREAMS Central 1 AOR = 3.44, 95% CI: 1.72, 6.91; DREAMS Central 2 AOR = 7.52, 95% CI: 3.20, 17.75; Other Districts AOR = 1.89, 95% CI: 1.01, 3.54). The odds were significantly higher in DREAMS Central 1 (*p* = 0.0430) and DREAMS Central 2 (*p* = 0.0375) than the odds in Other Districts. AGYW in DREAMS Central 1 and DREAMS Central 2 who experienced physical violence compared to those who did not, had higher odds of engaging in recent TS (DREAMS Central 1 AOR = 7.51, 95% CI: 2.10, 27.04; DREAMS Central 2 AOR = 2.58, 95% CI: 1.10, 6.04). The odds were significantly higher in DREAMS Central 1 than the odds in Other Districts (*p* = 0.0122) and were not significantly higher in DREAMS Central 2 compared to Other districts (*p* = 0.0976). AGYW in all regions who experienced emotional violence compared to those who did not, had higher odds of engaging in recent TS (DREAMS Central 1 AOR = 3.79, 95% CI: 1.94, 7.40; DREAMS Central 2 AOR = 3.03, 95% CI: 1.39, 6.61; Other Districts AOR = 2.26, 95% CI: 1.13, 4.51). The odds were significantly higher in DREAMS Central 1 than the odds in Other Districts (*p* = 0.0296) This difference was not significant for DREAMS Central 2 compared to Other districts (*p* = 0.0732).

**Table 5:** Interactions between violence and violence-related experiences, and geographic region and recent transactional sex^a^ among sexually active^b^ Ugandan girls and young women age 13–24 years old.

National				
	Unadjusted OR (95% CI)	P-value	Adjusted OR* (95% CI)	P-value
Witnessed violence in the home*Region Within group comparison of witnessing violence in the home (ever vs. never [ref])				
DREAMS Central 1	**2.32 (1.18–4.55)**	**0.0144**	**2.21 (1.12–4.34)**	**0.0229**
DREAMS Central 2	**2.44 (1.30–4.60)**	**0.0058**	**2.72 (1.39–5.30)**	**0.0035**
Other Districts	1.23 (0.61–2.50)	0.5571	1.24 (0.60–2.53)	0.5611
Witnessed violence in the community*Region Within group comparison of witnessing violence in the community (ever vs. never [ref])				
DREAMS Central 1	**3.61 (1.92–6.80)**	**< .0001**	**3.54 (1.81–6.96)**	**0.0003**
DREAMS Central 2	**3.52 (1.80–6.98)**	**0.0003**	**3.41 (1.62–7.19)**	**0.0013**
Other Districts	1.08 (0.59–1.97)	0.8039	1.21 (0.68–2.15)	0.5226
Sexual Violence*Region Within group comparison of sexual violence (ever vs. never [ref])				
DREAMS Central 1	**3.53 (1.86–6.71)**	**0.0001**	**3.44 (1.72–6.91)**	**0.0005**
DREAMS Central 2	**7.79 (3.39–17.91)**	**< .0001**	**7.52 (3.20–17.75)**	**< .0001**
Other Districts	**1.92 (1.03–3.58)**	**0.0395**	**1.89 (1.01–3.54)**	**0.0463**
Physical Violence*Region Within group comparison of physical violence (ever vs. never [ref])				
DREAMS Central 1	**8.29 (2.41–28.51)**	**0.0008**	**7.51 (2.10–27.04)**	**0.0021**
DREAMS Central 2	**2.67 (1.21–5.88)**	**0.0147**	**2.58 (1.10–6.04)**	**0.0294**
Other Districts	1.30 (0.61–2.80)	0.4954	1.46 (0.66–3.22)	0.3525
Emotional Violence*Region Within group comparison of emotional violence (ever vs. never [ref])				
DREAMS Central 1	**3.57 (1.94–6.58)**	**< .0001**	**3.79 (1.94–7.40)**	**0.0001**
DREAMS Central 2	**3.21 (1.59–6.49)**	**0.0012**	**3.03 (1.39–6.61)**	**0.0056**
Other Districts	**2.23 (1.15–4.33)**	**0.0181**	**2.26 (1.13–4.51)**	**0.021**

CI = confidence interval.

^a^Recent transactional sex is defined as transactional sex in the past 12 months.

^b^Sexually active is defined as having sex (vaginal, oral, or anal sex) in the past 12 months.

^c^Bolded results have odds ratio p-values less than 0.05.

*OR adjusted for: Early sexual debut, ever married, family wealth, education, and ever pregnant.

## Discussion

This study identifies individual characteristics, including witnessing violence and violence victimization, that are associated with recent TS to better understand patterns of risk for TS and HIV, and to inform strategic targeting of AGYW at risk of TS with programming to meet their unique HIV prevention needs. Numerous interesting relationships emerged, the most striking being the consistent and strong associations between exposure to violence and recent TS in high HIV prevalence DREAMS regions compared to the rest of Uganda. To our knowledge, this is the first study to assess factors associated with TS in targeted geographic locations in a single country or using data from a national survey on violence. This study provides evidence that localized data on risks associated with TS may provide nuanced programming insights.

The high prevalence of recent TS, a known HIV risk behavior, among sexually active AGYW in Uganda (14.2%) suggests a need to continue to strengthen efforts to address economic and social risk factors to help prevent young women from having to rely on TS [2]. Surveys with narrower definitions of TS, often using measures which may conflate TS as sex work [36], have found lower national prevalence estimates in Uganda (3.7%; [37]), while studies of specific populations in Uganda, including university students (15.2%), have recorded similar prevalence for lifetime participation in TS. Other estimates of TS in sub-Saharan Africa have ranged from 2.1% in a nationally representative survey in South Africa using a narrow definition of TS to 25% in Eastern Cape, South Africa in a study using a similar definition to the Uganda VACS [38]. The relatively high prevalence of recent TS among AGYW in DREAMS Central 1 (20.5%) and DREAMS Central 2 (19.2%) compared to Other Districts in Uganda (13.8%) points to important geographic differences in risk factors for HIV. HIV prevention and control efforts would benefit from reflecting on geographically localized patterns of drivers of the epidemic, which was the approach used to identify districts appropriate for DREAMS.

Two out of five (43.8%) AGYW who had recent TS had older partners, providing evidence that TS behaviors include age-disparate relationships, an important factor in HIV transmission, however more than half of TS partners occurred with closer age-matched partners. Conceptualizing TS as coercive due to power imbalances in age-disparate relationships may be valid for a proportion of AGYW. However, this is an incomplete portrayal of AGYW’s experiences and factors influencing TS among those engaging in TS with peers. That 79% of AGYW who had TS reported TS with a current or ex-romantic partner and 24% with a current or former spouse indicates that these relationships are complex, consistent with research indicating that many AGYW expect to participate in TS within a normal romantic partnership, even as a pathway to marriage [21,39].

### Socio-demographic factors associated with TS

In the Other Districts (not DREAMS Central 1 or 2), orphanhood was associated with higher odds of recent TS, aligning with previous research in which TS was more common among youth raised without one or both parents [27]. Low socioeconomic status in Other Districts and marriage/cohabitation in DREAMS Central 1 and Other Districts were negatively associated with TS, as was ever experiencing a pregnancy in Other Districts. The protective nature of marriage aligns with previous studies in which TS was less common among married AGYW and aligns with anthropological analyses suggesting that demonstrations of male provision and offering sexual access in return may be a part of the process toward establishing a union for some young people [39]. The negative association between family wealth and TS in Other Districts counters common narratives of TS among the most vulnerable AGYW. Low family wealth was not significantly associated with TS in DREAMS Central 1 or 2, potentially reflecting differences in access to TS exposure for the poorest members of communities in mostly rural Uganda. The proportion of AGYW in the lowest wealth tertile was higher in Other Districts (28.8%) than in DREAMS Central 1 (13.8) or 2 (12.8%). These findings further support the idea that TS among AGYW in Uganda is not necessarily a means to meet basic survival needs. Rather, these findings indicate that the underlying vulnerabilities leading to TS are more socially and psychologically complex than simple economics. In further considering potential economic motivation for TS, we note that most common items received in exchange for sex were money, favors or gifts, or food, suggesting that AGYW in Uganda do participate in TS to meet certain perceived needs. However, these findings indicate that even as a sub-set of AGYW may engage in TS toward meeting their *basic* needs (e.g. sex in exchange for food), most are not engaging in TS to survive. Rather, these findings indicate that the underlying vulnerabilities leading to TS are more socially and psychologically complex than simple economics. These findings may be better understood in light of Stoebenau et al’s [10] paradigms for transactional sex: ‘sex for material gain’, ‘sex for improved social status’, and ‘sex as an expression of love’. This summary of findings related to the motivations of AGYW to engage in TS provides insight into the perceptions of AGYW on why they engage in TS while this research may provide additional understanding of why AGYW have the motivations they do. Specific to Central Uganda, Stoebenau, 2019 found that it is socially acceptable to find a second partner if a primary partner is not providing adequate support and multiple other studies have provided evidence that material gain for increased social status as a motivator for TS.

Critically, our findings indicate that factors associated with TS differ somewhat across geographic regions or contexts. Unique patterns emerged in associations between TS for orphan status, marriage, and family poverty in Other Districts. These factors did not emerge as significantly associated with TS in the DREAMS Central 1 or 2 regions, indicating that factors associated with TS differ based on local context. We hypothesize that this trend would not be limited to the Ugandan context and that this analytic approach to assess potential determinants for TS regionally could provide important information for more targeted programming in other sub-Saharan African countries.

Early sexual debut was associated with recent TS in DREAMS Central 1 and 2, though not in Other Districts. Both early sexual debut and TS are associated with risk for HIV, though limited research has focused on interrelatedness among these two factors. Our findings suggest early sexual debut and TS are associated in areas with high HIV prevalence.

### Violence exposures and TS

Exposure to sexual violence and emotional violence by parents were both associated with increased odds of recent TS in all regions. Additional associations between TS and violence emerged in both DREAMS Central 1 and 2, with higher odds of TS for those who reported witnessing violence in the home or community in childhood along with lifetime sexual or emotional violence. In DREAMS Central 1, physical violence was also associated with six-fold increased odds of TS. The associations between violence and TS in this study expand upon previous work establishing links between violence and TS through varying mechanisms [15,16,27].

Although less is understood about specific pathways between exposure to violence and TS, childhood trauma resulting from such adverse childhood events as violence victimization or witnessing violence are well-established risk factors for negative mental and physical health outcomes including increased likelihood of engaging in sexual risk behaviors [40].

Fewer significant associations between violence and TS emerged in the Other Districts, though generally patterns reflected similar directional relationships. The interaction between violence and region further highlights the regionally specific relationship between violence and TS. Although DREAMS Central 1 and 2 are not the only regions with high HIV burden in Uganda, the complex contexts and social environments therein, including fatalism among the fishing communities in DREAMS Central 1, and the transience of populations in DREAMS Central 2, contribute to the HIV epidemic. Data on violence and TS in these focused geographic regions could be reflective of a clustering of many of the same risk factors that both contribute to and emerge as a result of the HIV epidemic. Given the geographic findings on the protective nature of specific social factors, future research could examine the relevance of social connectedness as a factor contributing to associations between violence and TS and could consider emotional dysregulation as a pathway between violence and TS, one that may be exacerbated by other complex factors in Central 1 and 2. These findings indicate that our understanding of the local context is critical to effectively respond to and prevent TS behavior and its role in the HIV epidemic.

TS is associated with a 50% increased risk of HIV among AGYW, making it a top-priority issue in HIV epidemic control [10]. TS prevention efforts have focused largely on relieving economic pressure and changing harmful gender norms [41]. The present findings indicate that factors associated with TS differ by context, suggesting interventions should be tailored to meet the needs of AGYW in their unique social environments. Targeted interventions must take into consideration patterns of violence that may impact AGYW’s decision making processes in addition to or even more than their economic vulnerability.

Considering the association between violence and TS and the protective association with talking to friends about important things and TS, efforts to reduce new HIV infections among AGYW in Uganda may benefit from approaches that prevent and respond to violence and promote social asset building. Evidence-based approaches including cash transfers, group savings and loan programs, and microfinance programs such as Empowerment and Livelihoods for Adolescents [42] could provide much needed support to AGYW facing socio-economic hardship with an aim to prevent TS. Access to such programming, tailored to meet the challenges AGYW face in their communities, can provide beneficiaries, their families, and their communities with the tools to prevent violence, and provide services to victims of violence as secondary prevention to address potential ongoing health issues. The Government of Uganda’s District Action Centers (DAC) hold promise in providing a safe drop-in location for children to seek help for violence and in ensuring the national Child Helpline (Sauti 116) is able to respond to reported cases of violence throughout the country by linking with DAC staff for quality case management.

Recognizing the high prevalence of emotional, sexual and physical violence among AGYW, there is an urgent need for rapid expansion of violence prevention and response programming in Uganda. Programming has increasingly incorporated parenting interventions, such as Parenting for Lifelong Health, implemented as ‘Sinovuyo’ in DREAMS programming in Uganda, which promote nurturing and positive parent-youth relationships and reduce physical and emotional violence by parents [42]. These findings provide evidence that efforts to increase positive parenting can prevent violence and in turn reduce risk for TS and HIV.

Witnessing violence in the home and in the community were associated with TS in both DREAMS Central 1 and 2. Evidence-based strategies that reduce youth exposure to violence in their homes and communities can ensure protective environments for young people and may reduce risk for TS and its negative health consequences.

Recognizing the strong associations between violence and TS, it is critical that interventions aim to decrease HIV risk associated with TS and incorporate early comprehensive violence prevention programming. This research adds evidence that witnessing violence and experiencing physical and emotional violence are important considerations, in addition to the previously recognized role of sexual violence as a driver of TS and HIV [43]. Curbing the HIV epidemic among AGYW may involve a broader approach to violence prevention, taking multiple forms of violence exposure into account. Home-grown evidence-based violence prevention programs endorsed in the INSPIRE technical package, including Raising Voices’ SASA! and Good Schools Toolkit can be scaled up quickly and efficiently with increased commitments from government, partners, and donors.

### Limitations

These findings are subject to several limitations. Because this analysis examined TS in the past 12 months and lifetime exposure to violence, the temporality of these experiences is unknown. We expect that most experiences of violence occurred prior to the past 12 months, but it is also likely that many participants experienced violence both recently and historically and that some participants only experienced recent violence. In Uganda, among females who experienced sexual violence prior to age 18, 83% had experienced multiple incidents of sexual violence. Similarly, the majority of those who experienced physical and emotional violence experienced multiple incidents of that type of violence (91% and 82%, respectively) [9]. The relationship between violence and TS is likely bi-directional, with those witnessing and experiencing violence at higher risk of TS and those engaging in TS at increased risk of violence victimization. Previous research among females in Central Uganda has identified high rates of IPV among women engaged in TS and noted the bi-directional nature of the relationship, though definitions of TS appear synonymous with sex work and the focus was on very localized populations [44]. Self-report of HIV status is another limitation of this study to consider, particularly since TS was negatively associated with HIV testing, therefore knowledge of HIV status in this population may be limited. The sample of AGYW who experienced transactional sex in the past 12 months was relatively small. Although we believe the analysis to be powered sufficiently to detect significant differences, or lack thereof, type-2 error may lead to false negatives. Related, an absence of association in some settings and not others may be due to lower prevalence of the factor in that setting. Some point estimates, such as the physical violence*region DREAMS Central 1 OR in Table 5 (AOR = 7.51, 95% CI: 2.10, 27.04), have wide confidence intervals indicating a less precise estimate and should be considered in the interpretation of the result. Finally, there are limitations to the generalizability of the findings. The Uganda VACS did not collect data on some of the most vulnerable AGYW, such as those who live in institutions, on the streets, those with disabilities or those unable to consent.

## Conclusions

Violence is strongly and consistently associated with TS, and patterns in prevalence and risk factors vary across geographic regions in Uganda. These findings provide evidence that negative life experiences, such as violence, may impact underlying drivers of TS, beyond perceived motivations, and that this may be geographically contextual. Given the high risk of HIV associated with TS, HIV epidemic control efforts intended to target TS must focus on comprehensive violence prevention as a primary prevention strategy for HIV. HIV prevention programming aimed at keeping AGYW HIV-negative, such as PEPFAR’s DREAMS initiative, incorporate prevention of violence and TS as key components in comprehensive HIV prevention packages which will contribute to achieving epidemic control.
